# A rare loss-of-function variant of ADAM17 is associated with late-onset familial Alzheimer disease

**DOI:** 10.1038/s41380-018-0091-8

**Published:** 2018-07-09

**Authors:** Daniela Hartl, Patrick May, Wei Gu, Manuel Mayhaus, Sabrina Pichler, Christian Spaniol, Enrico Glaab, Dheeraj Reddy Bobbili, Paul Antony, Sandra Koegelsberger, Alexander Kurz, Timo Grimmer, Kevin Morgan, Badri N. Vardarajan, Christiane Reitz, John Hardy, Jose Bras, Rita Guerreiro, Rudi Balling, Jochen G. Schneider, Matthias Riemenschneider, AESG, Celeste Sassi, J. Raphael Gibbs, Dena Hernandez, Keeley J. Brookes, Tamar Guetta-Baranes, Paul T. Francis, Michelle K. Lupton, Kristelle Brown, John Powell, Andrew Singleton

**Affiliations:** 1 Department of Psychiatry and Psychotherapy, Saarland University Hospital, Saarland University, Homburg, Germany; 2 Luxembourg Centre for Systems Biomedicine (LCSB), University of Luxembourg, Esch-sur-Alzette, Luxembourg; 3 Institute for Systems Biology, Seattle, WA USA; 4 Department of Psychiatry and Psychotherapy, Klinikum Rechts der Isar, TU-Muenchen, Munich, Germany; 5 School of Life Sciences, Queens Medical Centre, University of Nottingham, Nottingham, UK; 6 Taub Institute for Research on Alzheimer’s Disease and the Aging Brain, College of Physicians and Surgeons, Columbia University, New York City, NY USA; 7 Department of Neurology, College of Physicians and Surgeons, Columbia University, New York City, NY USA; 8 Gertrude H. Sergievsky Center, College of Physicians and Surgeons, Columbia University, New York City, NY USA; 9 Department of Epidemiology, Mailman School of Public Health, Columbia University, New York City, NY USA; 10 Department of Molecular Neuroscience, Institute of Neurology, University College London, London, UK; 11 UK Dementia Research Institute at UCL (UK DRI), London, UK; 12 Department of Medical Sciences, Institute of Biomedicine-iBiMED, University of Aveiro, Aveiro, Portugal; 13 Department of Experimental Neurology, Center for Stroke Research Berlin (CSB), Charité - Universitätsmedizin Berlin, Corporate Member of Freie Universität Berlin, Humboldt-Universität zu Berlin, and Berlin Institute of Health, Berlin, Berlin, Germany; 14 Laboratory of Neurogenetics, National Institute on Aging, National Institutes of Health, Bethesda, MD USA; 15 Translation Cell Sciences-Human Genetics, School of Life Sciences, Queens Medical Centre, University of Nottingham, Nottingham, UK; 16 Brains for Dementia Research Resource, Wolfson Centre for Age Related Diseases, King’s College London, London, UK; 17 QIMR Berghofer Medical Research Institute, Brisbane, Queensland, Australia; 18 King’s College London, Institute of Psychiatry, Psychology and Neuroscience, London, UK

**Keywords:** Neuroscience, Molecular biology, Genetics

## Abstract

Common variants of about 20 genes contributing to AD risk have so far been identified through genome-wide association studies (GWAS). However, there is still a large proportion of heritability that might be explained by rare but functionally important variants. One of the so far identified genes with rare AD causing variants is *ADAM10*. Using whole-genome sequencing we now identified a single rare nonsynonymous variant (SNV) rs142946965 [p.R215I] in *ADAM17* co-segregating with an autosomal-dominant pattern of late-onset AD in one family. Subsequent genotyping and analysis of available whole-exome sequencing data of additional case/control samples from Germany, UK, and USA identified five variant carriers among AD patients only. The mutation inhibits pro-protein cleavage and the formation of the active enzyme, thus leading to loss-of-function of ADAM17 alpha-secretase. Further, we identified a strong negative correlation between *ADAM17* and *APP* gene expression in human brain and present in vitro evidence that *ADAM17* negatively controls the expression of *APP*. As a consequence, p.R215I mutation of ADAM17 leads to elevated Aß formation in vitro. Together our data supports a causative association of the identified *ADAM17* variant in the pathogenesis of AD.

## Introduction

The central theory on Alzheimer disease (AD) pathogenesis is the amyloid cascade hypothesis with amyloid ß peptides (Aß), formed from the ß-amyloid precursor protein (APP) as etiologic agent [1]. The importance of APP metabolism was confirmed by genome-wide association studies (GWAS) and reports of rare gene variants in patients with familial AD [2]. Historically, the majority of mechanistic studies was focused on APP processing by alpha-, beta-, and gamma-secretase to illuminate the cellular pathways of Aß production as pharmaceutical targets [1]. In addition to APP metabolism, the so far known AD risk genes are involved in regulation of the immune system, cholesterol metabolism, cytoskeleton development, and other. However, there is still a large proportion of missing heritability that might be explained by rare but functionally important variants.

We now report a rare single-nucleotide variation (SNV) of the APP alpha-secretase *ADAM17* in a family with autosomal-dominant late-onset AD that results in loss of enzyme function. We further identified a strong negative regulatory effect of ADAM17 on APP expression in human brain. This novel function in the newly identified *ADAM17* variant results in an upregulation of *APP* gene expression and consequently elevated Aß formation in vitro.

ADAM17, also known as tumor necrosis factor-alpha converting enzyme (TACE) is a sheddase with over 80 so far described substrates acting in different physiological processes [3]. It was initially described as the enzyme cleaving membrane bound tumor necrosis factor (TNF)-alpha precursor to a soluble form [4]. Later on, ADAM17 was identified as the first alpha-secretase candidate processing APP [5], followed by ADAM9 and ADAM10 [6, 7]. Rare variants of *ADAM10* have previously been identified in late-onset AD patients [8].

Recently, ADAM17 was also identified as main sheddase of TREM2. Rare genetic variants of *TREM2* considerably contribute to AD risk [2].

## Methods

### Clinical characterization of the AD family

**Fig. 1:**
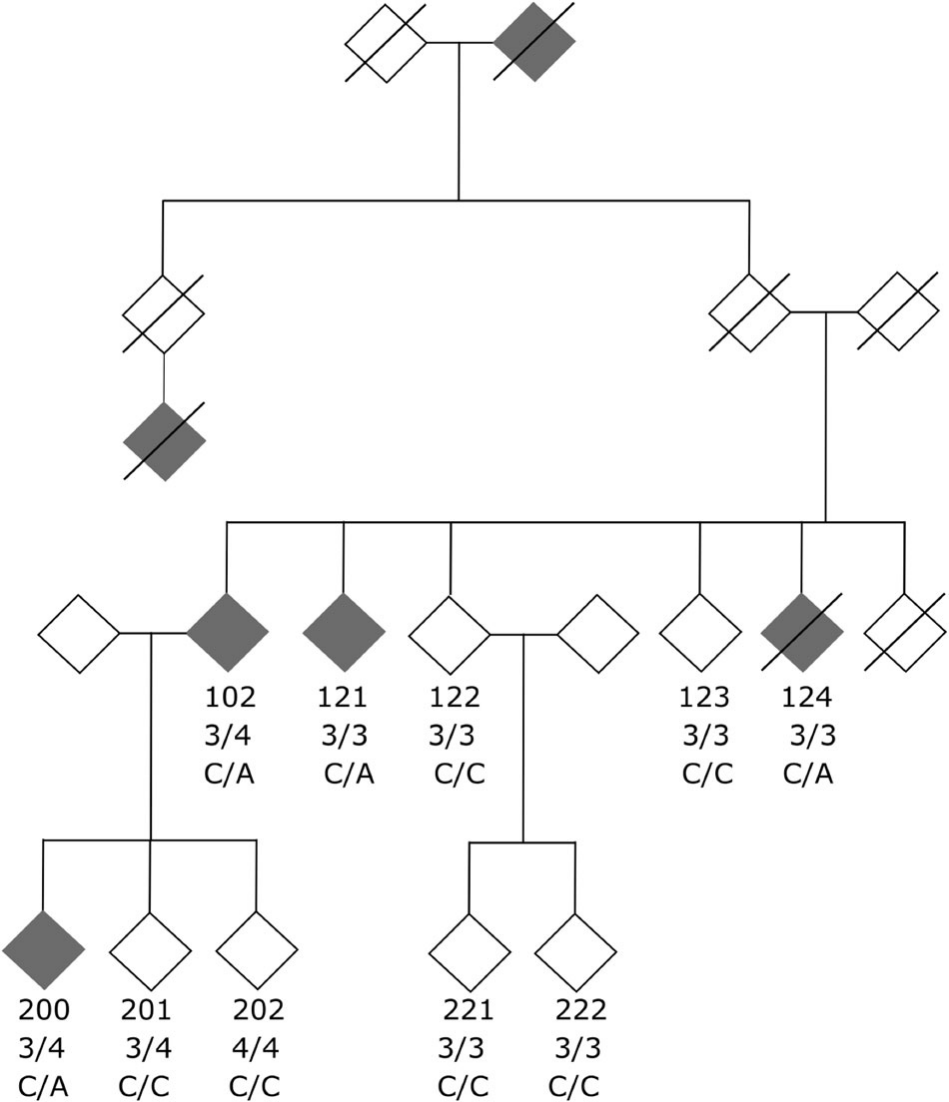
Pedigree of AD family. The affected and unaffected individuals are filled in gray and white, respectively. The *APOE* status is given beneath individual identifiers. Carriers of the heterozygous variant (rs142946965) are indicated as C/A and carriers of the wild-type *ADAM17* version are indicated as C/C

All participants gave written informed consent for the clinical evaluation and genetic analysis of leukocyte DNA. Clinical phenotyping, whole-genome sequencing (WGS) and genetic analysis was approved by the ethical review committee of the Bavarian medical association and by the Ethics Review Panel of the University of Luxembourg. The pedigree chart is given in Fig. 1.

The four affected patients with AD (patients 200, 102, 121, and 124) had a reported age of onset of 60 (patient 200), 69 (patient 102), their *APOE* status was ε3/4, ε3/4, ε3/3, and ε3/3, respectively. For patients 121 and 124, age of disease onset was not reported, age of examination at clinic was 87 and 70, respectively. Among patients unaffected by AD, the patients 122 and 202 had symptoms of depression; and patient 123 was diagnosed with Parkinson’s disease. Patient 200 was diagnosed with AD one year after onset of symptoms with a MMSE score of 28/30. Psychometric testing (CERAD-NAB) revealed impairment in verbal learning and recognition as well as in delayed verbal and non-verbal recall. Cranial MRI was without abnormalities, in particular no atrophy, FDG-PET showed markedly reduced tracer uptake temporal/ parietal, and occipital (sparing of the primary visual cortex) and posterior cingulate Gyrus (right > left). One year after first examination clinical and imaging data showed a considerable progression, MMSE dropped to 26/30 and CERAD-NAB showed impairment in verbal fluency, verbal learning and recognition, verbal and non-verbal delayed recall, and visuoconstruction; cranial MRI revealed cortical atrophy temporal and parietal as well as hippocampal atrophy (right > left). The patient died 10 years after onset of disease symptoms. No concomitant diseases, drug abuse, or medications were reported. Neurological examination was normal and revealed no parkinsonian symptoms. Family members 201, 202, 221, and 222 were unaffected, but too young for a final diagnosis at the time of consulting with an age of examination at clinic of 55, 49, 55, and 52, respectively. The two non-affected individuals (122 and 123) had an age of examination at clinic of 81 and 74, respectively. Blood samples were taken from ten family members. DNA was extracted from leukocytes using standard procedures. Affected family members were pre-screened and negative for mutations in *APP*, *PSEN1*, and *PSEN2*.

### Genome sequencing and analysis

Whole-genome sequencing (WGS) was performed by Complete Genomics Inc. (CG, Mountain View, CA, USA) using paired-end, nanoarray-based sequencing-by-ligation technology [45]. Six individuals of the family were selected for WGS (Fig. 1 and Suppl. Figure 2B), two affected (102 and 200) and four unaffected (122, 123, 201, and 202) family members. After quality control, DNA samples were sent to CG for sequencing. The sequencing data was processed using the Standard Sequencing Service pipeline version 2.0. Sequencing reads were mapped against NCBI Build 37.

### WGS variant prioritization pipeline

Only single-nucleotide polymorphisms (SNPs) and small indels (insertions, deletions and block substitutions up to a size of about 50 nt) were used for further analyses. As input for our WGS family analysis pipeline [9] we first combined all variants from the genomes of the sequenced family members into the union of variants using the CG Analysis^TM^ Tools (*CGATOOLS v.1.5*) *listvariant* command and the CG ‘var’ files as input. We used *CGATOOLS testvariant* command to test each genome for the presence of each allele at every variant position from the union set of variants. The average fully called genome fraction, defined as the fraction of reference bases, for which alleles could be called, was 96.4%, of which 99.1% were covered at least ×5 and 74.9% with at least ×30. On average, 99.6% of exons as defined by NCBI RefSeq 37.2 were fully called with at least ×5 and 86% with at least ×30. A statistical summary for all samples is given in Suppl. Table 3. We detected on average 3,441,899 SNVs and 606,446 indels and substitutions per genome. The fraction of novel variants, here defined as variants not previously found in dbSNP build 135, was on average 0.03 for SNPs and 0.27 for indels and substitutions. Relationship estimation [10] confirmed all relationships given the original pedigree information. We removed variants that were not called as high-quality calls (VQHIGH) in at least one individual. We used ISCA version 0.1.9 [11] to search for shared haplotype blocks between the two cases in the families. The shared regions are shown in Suppl. Figure 1. Variants were then filtered for the shared haplotype regions. By applying a dominant inheritance model, we filtered for variants that were called heterozygous (or have no-calls) in cases and were not called in any control genome. The remaining variants were annotated using ANNOVAR [12] (version 2015Mar12) using the NCBI RefSeq release 60 and the Ensembl release 74 genome annotations and multiple annotation databases available from the ANNOVAR webpage. We further filtered for exonic regions and excluded synonymous variants. Candidate variants should be rare in the general population (here defined by an allele frequency equal or <1%). Allele frequencies were determined from the European American population of the 1000 genomes project, the European NHLBI ESP exomes, and the Non-Finnish European population from the ExAC project as well as in the control data set CG69 provided by CG. Since certain genes, like mucins and olfactory receptors, are known to have higher mutation rates, variants in such genes were excluded. The list of commonly mutated genes (CMDGenes) is given in Suppl. Table 4. To narrow down the list of candidate variants, we filtered for genes that are known to be expressed in brain tissues. The brain-expressed genes were defined using the data set curated by [13], and genes expressed in brain regions from the Human BodyMap 2.0 project processed with the GeneAtlas tool (http://caballero.github.io/GeneAtlas/). We further filtered for pathogenicity by considering either loss-of-function variants (indels, stop gain, and splice-site variants) or nonsynonymous mutations predicted to be deleterious by at least 5 out of 7 pathogenicity scores (SIFT, Polyphen2_HDIV, Polyphen2_HVAR, LRT, Mutationtaster, PROVEAN, MetaSVM, fathmm-MK_coding_pred, as given by dbNSFP3.0a [14]) and two out of four conservation scores (DAMN, CADD, GERP, SiPHY). A list of candidate genes related to AD and neurodegenerative diseases were collected from various GWAS studies in dbGAP, the Alzgene database [15] and the Genotator tool [16]. In total, we collected a final list of 2352 candidate genes related to neurodegenerative diseases (Suppl. Table 5, NDGenes). The variants were examined using the candidate gene list and the KGGseq tool [17]. Each variant was tested if the affected gene co-occurred with any candidate gene within a certain pathway or if it was connected within a first neighborhood to one of the neurodegenerative candidate genes within a protein–protein interaction network using the STRING database [18]. The whole filtering strategy is shown in Suppl. Figure 2A.

### Genetic and linkage analysis

For linkage analysis, high-quality SNP positions (complete call rate over all individuals (VQHIGH status in CG var files) were extracted from the WGS data. Using variants in linkage disequilibrium (LD) for the linkage analysis might lead to false positive results. Hence, the variants with high LD were removed by using PLINK 1.9 [19]. Further thinning of variants was performed by using mapthin software [20]. A set of 2000 variants per chromosome along with the identified variants segregating with the disease through the pedigree were used to check for genotype errors and Mendelian inconsistencies using MERLIN [21] and were subsequently removed if they were identified as errors. Remaining variants were used for linkage analysis and their genomic positions were linear interpolated based on hapmap genetic map (2011-01_phaseII_B37). MERLIN was used to perform both haplotyping and multipoint parametric linkage analysis with a rare dominant disease model with a disease frequency of 0.0001 and penetrance of 0.0001,1.0,1.0. Haplotyping results were visualized using Haplopainter [22] (Suppl. Figure 2B-C). Using the R package ‘paramlink’ [23] we calculated the power of the pedigree given as the maximal LOD score (1.20412) under an autosomal-dominant inheritance model and 10,000 simulated markers. Relationship detection between all individuals was performed using the software GRAB [10]. Validation of results by Sanger sequencing was carried out by Seq IT GmbH, Kaiserslautern, Germany.

### Expression correlation analysis of ADAM17 and APP in human brain tissue

Correlation analysis of *APP* and *ADAM17* gene expression was carried out using a sample set obtained from fresh frozen post-mortem prefrontal cortex of 201 AD samples and 83 cognitively healthy controls, provided by The Netherland Brain Bank (NBB). A second sample set used for independent validation of results contained 57 fresh frozen post-mortem human temporal cortex samples (33 AD samples and 24 cognitively healthy controls) provided by Brain Bank Munich (MUC). Full sample characteristics are given in Suppl. Table 2. Total RNA was extracted from human brain tissue using either miRNeasy Mini Kit or RNeasy Lipid Tissue Mini Kit (Qiagen) according to manufacturer’s protocol including on-column DNA digestion with RNase free DNase Set (Qiagen). Total RNA was amplified and labeled with biotin using Ambion® Illumina® TotalPrep™ RNA Amplification Kit (Ambion) according to manufacturer’s protocol. cRNA was eluted in 50 μl nuclease-free water and precipitated using ammonium acetate (NH_4_OAc) when concentration was <150 ng/μl. Direct hybridization of 750 ng biotin labeled cRNA to Illumina® Sentrix BeadChips HumanHT-12 v4 (Illumina) was conducted according to manufacturer’s protocol. Whole-genome gene expression analysis incorporated 47,231 markers per sample including coding as well as non-coding transcripts based on Human RefSeq 38 (NCBI RefSeq database) and supplementary UniGene content. Processed BeadChips were scanned using the HiScan (Illumina). In the first quality check step, background subtraction, and quantile normalization were executed with GenomeBead Studio Module for gene expression. Markers with a detection *p*-value ≥ 0.05 were considered as no-call. Call rates for each marker were calculated separately in both groups (AD vs. control). Markers with a call rate <90 % in both groups were considered as non-informative and were excluded from further statistical analysis. Signal intensities were log_2_ transformed for further analysis. Correlation between the expression of *ADAM17* and *APP* was tested using the linear model fit function (lm) in the R package. *p*-value of the coefficient (slope of the fit) and adjusted *R*^2^ were reported. Correlation between *ADAM17* and *ADAM17* target gene expression was tested using the correlation test in R.

Additionally, the MERAV database and analysis tool [30] was used to examine gene expression correlations between *ADAM17* and *APP* in non-brain and brain tissues from public databases like ArrayExpress, GEO and the human body index. MERAV is a tool for comparing gene expression across different human tissues and cell types. The quantile normalized log-transformed expression values for the four genes for normal non-brain (502) and normal brain tissues (186) were downloaded and Pearson correlation between gene expression for all gene pairs were calculated using tool cor.test in R. Additionally, we tested the significance of the difference between correlations coefficients obtained for the non-brain and brain tissues using the R library psych and the one-sided paired r test.

### Generation of *ADAM17* variants

*ADAM17* cDNA was derived from clone HsCD00342307 (Harvard Medical School). Wild-type *ADAM17* was amplified and digested with KpnI and BamHI primers (for primer sequences see Suppl. Table 7), and ligated into pCDNA3.1(+) (Invitrogen # V790-20). Site-directed mutagenesis of *ADAM17* was done via PCR-driven overlap extension (PMID 17446874). For p.R215I and p.E406A mutagenesis primer sequences are given in Suppl. Table 6. Hybridization of 5′ and 3′ portions of *ADAM 17* variants was used for the amplification of full length *ADAM17* variants. For subcloning into pcDNA3.1(+) the full length *ADAM17* variants were enriched via PCR amplification using the primers KpnI_f and BamHI_r. Digestion and ligation steps were done as described above for wild-type *ADAM17*. Bacterial cultures with *ADAM17* constructs were grown at 24 °C for 48 h.

### Cell culture

SH-SY5Y cells stably expressing human APP_695_ carrying the Swedish double mutation (in pcDNA3.1, selection with 0.3 mg/ml hygromycin) were kindly provided by Tobias Hartmann and Marcus Grimm (Saarland University). SH-SY5Y cells were stably transfected with expression constructs for *ADAM17* variants (in pcDNA3.1, selection with 500 μg/ml geneticin). Cells were grown in DMEM with selection antibiotics and 10% fetal calf serum (FCS). For rescue experiments, cells were supplied with media containing 100 nM TAPI-0 (SantaCruz) or equal volume of DMSO as control. Medium was changed every second day, cells were checked for mycoplasma contamination.

### Protein and gene expression analyses in cells

The amounts of ADAM17 and APP in cellular lysates were determined by standard Western blotting techniques. Antibodies directed against ADAM17 or APP were obtained from commercial suppliers (ADAM17/TACE C15 and H-300, SantaCruz; APP 6E10, Covance). For lysis, cells were washed with ice-cold PBS and resuspended in ice-cold standard RIPA buffer containing 10 mM 1,10 phenantroline (Sigma-Aldrich) and protease inhibitor cocktail (Complete, Roche). Blots were developed using the SuperSignal West Femto Chemiluminescent Substrate (Pierce) and analyzed using the Peqlab FUSION XPress Imaging System. Beta-Actin or Alpha-Tubulin was analyzed as loading control. For statistical analysis, signals were normalized to loading controls and statistical significance was determined by non-parametric Kruskal–Wallis test and Dunn's multiple comparison test using Prism software (version 6.0d; Graphpad). Multiplicity adjusted *p*-values are given. Only relative signals (to control) were combined from different blots and all samples represent biological replicates.

Secreted Aβ_40_ and Aβ_42_ was quantified in cell culture media using commercial ELISA kits (EZBRAIN Aß_40_ and Aß_42_ Brain ELISA Kit, respectively Millipore). For ELISA, culture media was removed, cells were washed with PBS, then incubated in fresh medium containing 1% FCS for 48 h. After incubation, medium was collected, spun down and supernatant was used for measurements. Statistical significance of data was determined by ANOVA with Fisher's LSD test for pairwise comparisons using Prism software (version 6.0d; Graphpad).

Total RNA from cells was prepared using miRNeasy Mini Kit (Qiagen) according to manufacturer’s protocol. High-Capacity cDNA Reverse Transcription Kit (Applied Biosystems) was used for cDNA synthesis. Levels of APP mRNA expressed in SH-SY5Y cells were determined by quantitative RT-PCR using the Applied Biosystems 7500 Fast Real-Time PCR System. Samples were measured in triplicates, and calculations were performed using the Prism software (Graphpad, version 6.0d). Values for *APP* were normalized to 18 S expression determined in parallel in the respective samples. Fold changes were calculated by applying the 2^-ddCT method.

## Results

### Identification of an ADAM17 variant associated with familial AD

We identified an SNV of *ADAM17* co-segregating with the disease in a family with an autosomal-dominant pattern of inheritance of late-onset AD. Affected family members lacked pathogenic mutations of known AD genes. Whole-genomes of six family members (two affected and four unaffected, Fig. 1) were sequenced and analyzed by prioritizing rare, potential damaging variants that segregated with the disease. We searched for segregating variants by applying an autosomal-dominant inheritance model. In total 7,009,082 variants (SNPs and indels) different from the reference genome were identified in at least one family member. After strict quality filtering, 6.2 million variants remained. Due to the pedigree structure, we filtered for shared haplotype regions (Suppl. Figures 1 and 2A) between the two affected individuals (*n* = 3,903,193) and autosomal-dominant mode of inheritance (*n* = 187,733) filtering for heterozygous variants in the two affected and homozygous wild-type genotypes in the unaffected individuals 122 and 123. To further narrow down the list of variants, we screened for rare variants (*n* = 18,597) with predicted exonic defects (*n* = 64). After filtering out commonly mutated (*n* = 51) and variants in genes that are not brain-expressed (*n* = 25), we prioritized the remaining variants according to their predicted pathogenicity and conservation (*n* = 11, Suppl. Figure 2A, Suppl. Table 1). Only two out of the nine candidate variants were known relevant candidate genes (*ADAM17* and *ADRA2A*). Additionally, we performed linkage analysis for the pedigree using MERLIN [21]. Only two variants (*ADAM17* and *DDHD2*) fall into a region that showed the maximal LOD score (1.20) for this pedigree as calculated with ‘paramlink’ [23] (Suppl. Figure 2B-C). The *ADAM17* variant (NM_003183:exon6:c.G644T:p.R215I; rs142946965) was the only variant in an AD relevant gene predicted pathogenic by 7 out of 8 prediction programs (SIFT, Polyphen (HDIV/HVAR)), LRT, MutationTaster, Provean and FATHMM), conserved by three out of four conservation scores (DAMN, GERP, SiPHy) and additionally located within the maximal LOD score region of the pedigree. The presence of the heterozygous *ADAM17* variant (rs142946965) in the two affected family members was confirmed by Sanger sequencing and identified in two additional affected family members (121, 124, Fig. 1) that have not been analyzed by whole-genome sequencing (WGS). Also, the absence of the variant was verified in the four non-affected family members and in the non-affected family members 221 and 222 (Fig. 1) that have not been analyzed by WGS. The information generated by Sanger sequencing of additional family members results in a maximal LOD score of 1.81 as calculated with ‘paramlink’.

Together, our genetic approach including WGS, confirmation, and extension by Sanger sequencing identified the SNV rs142946965 of *ADAM17* to co-segregate with AD cases among the analyzed family members.

To further explore the possible relevance of the rs142946965 variant in AD we genotyped a German cohort (*N* = 1045: 969 cases, 76 controls) and analyzed this variant in exome sequencing data from a UK cohort (*N* = 571: 331 cases, 240 controls), and a cohort from the USA (Washington Heights-Hamilton Heights-Inwood Community Aging Project; WHICAP; *N* = 3834: 1567 cases, 2267 controls). The variant was not present within the control subjects. The same was true for an additional cohort of 468 unrelated Caucasian individuals of age 60 or higher from several UK and US brain banks who did not have a neurodegenerative disease diagnosis or disease-associated neuropathology at the time of death (Healthy exomes database, version 1.0) [24]. We identified five additional heterozygous variant carriers among AD subjects, one in each of the German and UK cohort, and three in the US cohort. The German variant carrier had an age of disease onset of 79 and the *APOE* status was ε2/3. The British variant carrier had an age at death of 92, with a hypothesized onset of disease at the age of 84 years, the *APOE* status was ε3/4. The three variant carriers in the US cohort had an age of disease onset of 85, 85, and 90, and one patient carries the *APOE* ε4 allele. All three US variant carriers were of Hispanic ethnicity.

Subsequent screening of genetic databases revealed an ultra-rare occurrence of the heterozygous rs142946965 variant in the European population (ESP6500_ea: 0.000077, 1000g_eur: 0.000199681, ExAC_NFE: 0.0001049) and no presence in any non-European populations. In the gnomAD database (http://gnomad.broadinstitute.org) from February 2017 the variant was present in 9 out of 138,390 individuals (allele frequency of 0.0000325), only in the heterozygous form and only present in the non-Finnish European population.

### The p.R215I mutation affects pro-protein convertase cleavage of ADAM17

A disintegrin and metalloprotease family (ADAM) members are multidomain proteins synthesized as latent precursors that are converted to mature, active enzymes by pro-protein convertase [25]. Enzymatic activity is inhibited via a cysteine switch mechanism forming a complex between a cysteine residue within the pro-domain and the zinc ion in the active center of the catalytic domain [26]. Activation of ADAMs requires proteolytic cleavage of the pro-domain. Pro-ADAM17 is activated in the trans-Golgi-network through furin cleavage at the last four amino acids preceding the catalytic domain [27]. The identified rs142946965, R215I variant of *ADAM17* causes a missense mutation changing positively charged, polar arginine to the non-polar, hydrophobic amino acid, isoleucine. The change is located at position 215 (corresponding to the canonical sequence of human ADAM17), right next to the pro-protein convertase cleavage motif (spanning positions 210–214 [28]), suggesting a possible interference with pro-protein convertase binding to ADAM17. To analyze possible effects of the mutation on ADAM17 biochemical function, we generated neuroblastoma (SH-SY5Y) cell lines stably over-expressing the newly identified variant of ADAM17 (p.R215I). In addition, we generated SH-SY5Y cells over-expressing human, wild-type ADAM17 (wild-type), and a catalytically inactive/dominant-negative form of ADAM17 (p.E406A), known from literature [29].

**Fig. 2:**
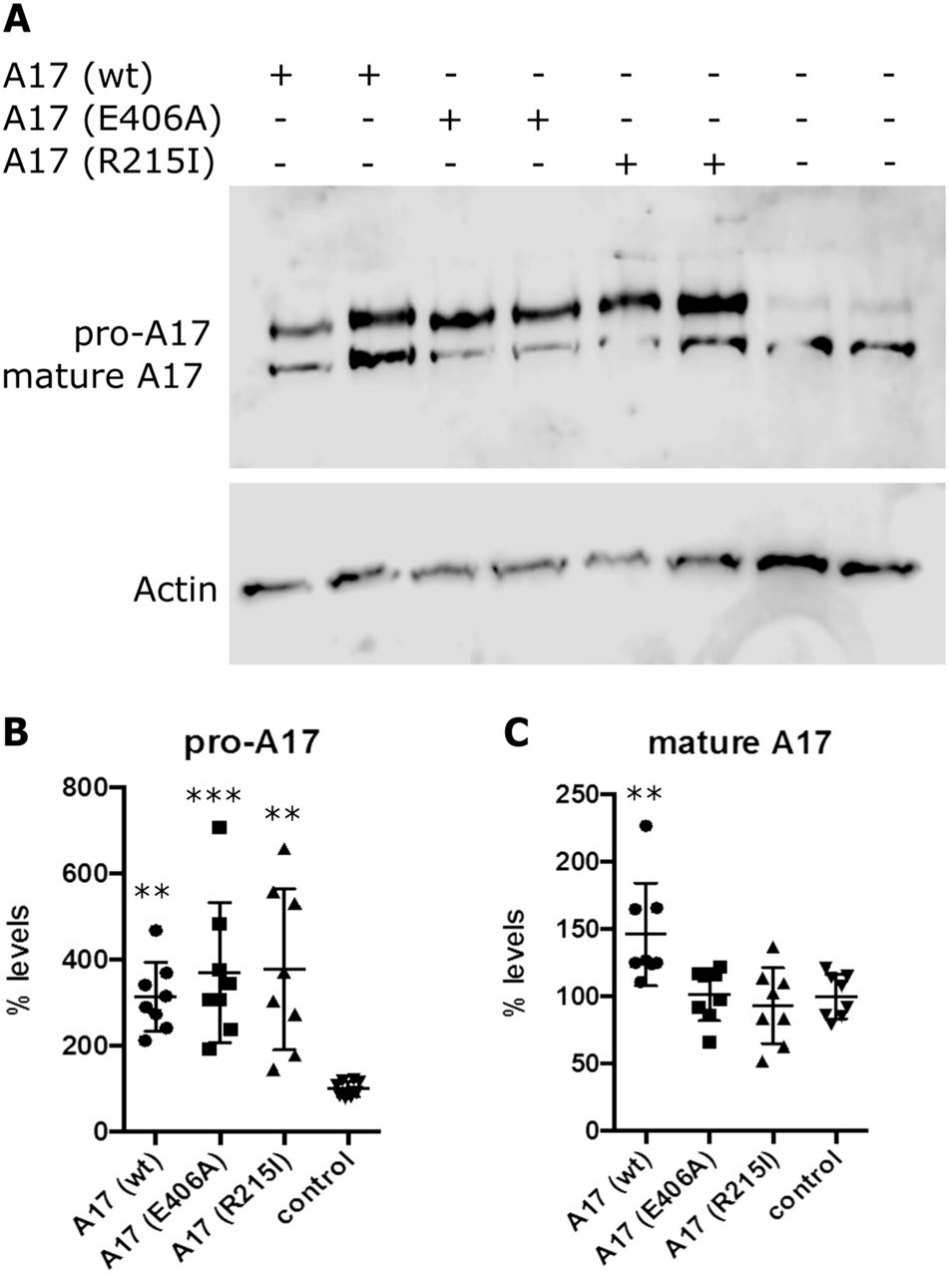
The R215I variant affects maturation of ADAM17. **a** Western blot showing ADAM17 (A17) protein expression in SH-SY5Y cells, over-expressing wild-type (wt) ADAM17, a dominant-negative form (E406A), or the newly identified AD variant of ADAM17 (R215I). The parental cell line was analyzed as control, actin-beta was analyzed as loading control (Actin). **b**, **c** Quantification of pro-ADAM17 signals shows significant upregulation in cell lines over-expressing ADAM17 variants (*n* = 8; p(wt) = 0.0057; p(E406A) = 0.0009; p(R215I) = 0.0014), respectively, as compared to the parental cell line (control). Quantification of mature ADAM17 signals only shows significant upregulation in cell lines over-expressing wild-type ADAM17 (*n* = 8; p(wt) = 0.0092), as compared to the parental cell line (control). Asterisks indicate significance. Error bars indicate mean with SD

Over-expression of ADAM17 in cells resulted in significant upregulation of total and pro-ADAM17 protein levels as compared to the parental cell line (control). This was true for all three variants, p.R215I, p.E406A, and wild-type ADAM17 (Fig. 2a–c). There was no significant difference between the p.R215I variant, the p.E406A variant and the wild-type variant of pro-ADAM17 levels. In contrast, levels of the shorter, mature form of ADAM17 lacking the pro-domain, were significantly upregulated exclusively in cells over-expressing wild-type ADAM17, but not altered in cells over-expressing p.R215I or p.E406A variants, as compared to control cells, respectively (Fig. 2a, c). These results demonstrate that as previously described elsewhere for the inactive p.E406A variant, pro-protein convertase cleavage is severely impaired in the newly identified ADAM17 variant (p.R215I), resulting in a lack of mature, catalytically active ADAM17 molecules.

### ADAM17 inhibits APP expression in vitro

Three members of the ADAM family, ADAM9, ADAM10, and ADAM17, show APP alpha-secretase activities [5–7]. The alpha-secretase initiates the non-amyloidogenic pathway by cleaving APP within the beta-amyloid sequence, which precludes the formation of beta-amyloid and further generates the neurotrophic and neuroprotective soluble N-terminal fragment of APP (sAPPα).

To analyze the effect of ADAM17 variants on Aß formation, we created SH-SY5Y cell lines over-expressing both human APP and ADAM17 (wild-type, p.E406A, or p.R215I variants, respectively). Maturation of mutated ADAM17 was again reduced in these cells (data not shown).

**Fig. 3:**
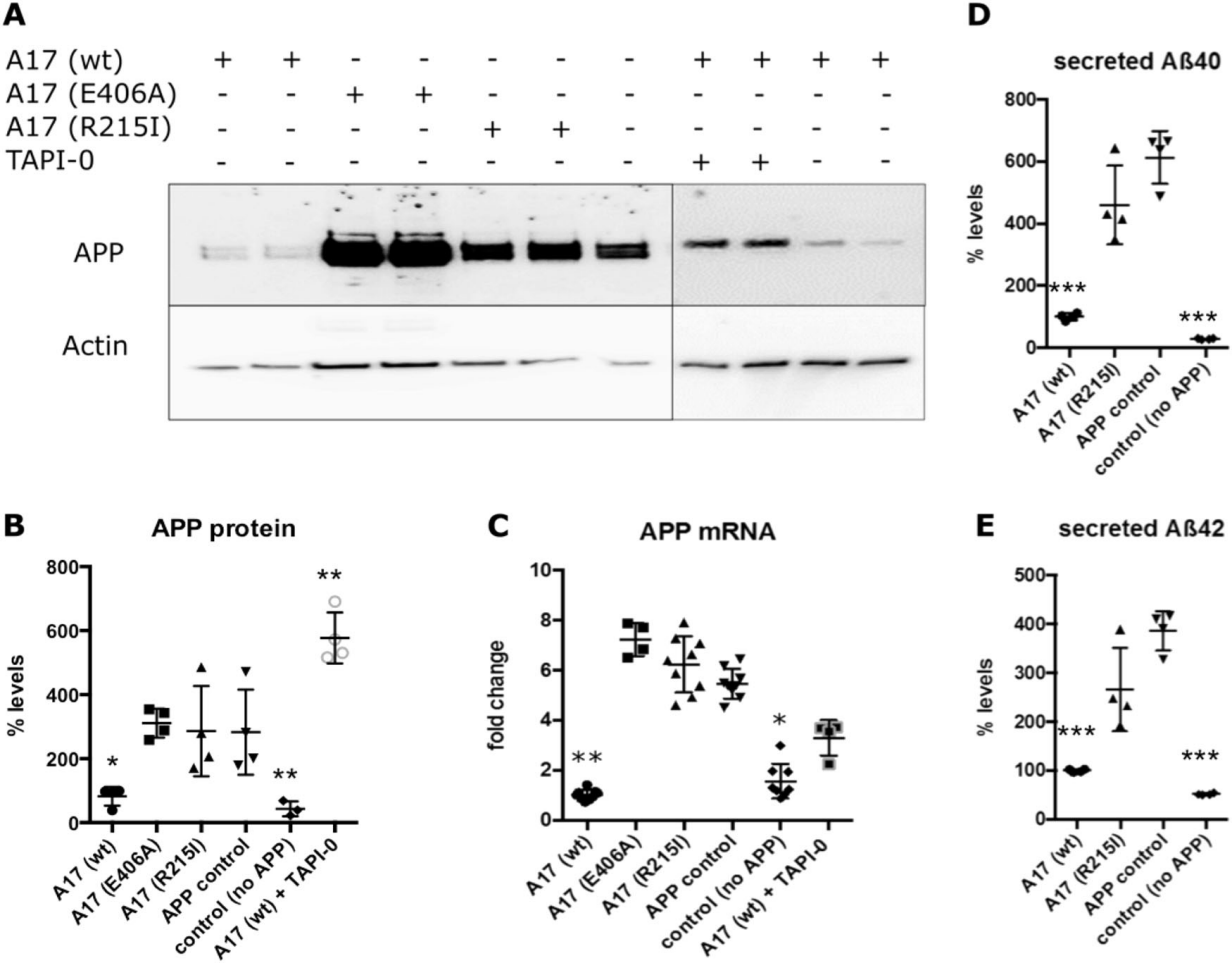
APP expression is downregulated by ADAM17. **a** Western blot showing total APP protein expression in lysates of cells over-expressing APP and respective variants of ADAM17 (A17). **b** Quantification of total APP protein signals shows significant downregulation in cells over-expressing wild-type (wt) ADAM17 and APP as compared to cells only expressing APP (APP control). No significant change was observed in cells expressing ADAM17 E406A and R215I variants, respectively, as compared to APP control cells. Rescue of APP expression was observed in A17 wt cells after treatment with ADAM inhibitor TAPI-0. **c** APP gene expression is significantly downregulated in cells over-expressing A17 wt and APP as compared to APP control cells (*n* = 9, p(wt) = 0.001). Rescue of APP expression was observed in A17 wt cells after treatment with ADAM inhibitor TAPI-0 (*n* = 3). APP was also significantly downregulated in the parental cell line that did not over-express APP (*n* = 9, p = 0.0341). **d** ELISA of Aß_40_ or **e** Aß_42_ in media of SH-SY5Y cells over-expressing APP shows significant downregulation of both Aß forms in A17 (wt), (p(Aß40) < 0.0001; p(Aß42)) = 0.0007; *n* = 4) cells as compared to APP control cells. For **b** to **e**: Asterisks indicate significance. Error bars indicate mean with SD

We first measured total APP protein and gene expression in ADAM17 and APP transgenic SH-SY5Y cell lines. Surprisingly, APP protein and gene expression were significantly reduced in cells expressing wild-type ADAM17 as compared to control cells expressing mutated forms of ADAM17, or APP only (Fig. 3a–c). Downregulation of APP gene expression in cells expressing wild-type ADAM17 was also observed in cells expressing endogenous APP (Suppl. Figure 3). In accordance with the observed reduction of APP expression by wild-type ADAM17, Aß40, and Aß42 secretion as measured by ELISA was upregulated in cells expressing the R215I variant as compared to cells expressing wild-type ADAM17 (Fig. 3d, e).

To analyze whether the observed downregulation of APP expression could be rescued by ADAM17 inhibition, we treated SH-SY5Y cells expressing both, APP and wild-type ADAM17, with a metalloprotease inhibitor (TAPI-0). Treatment of cells with 100 nM TAPI-0 inhibitor for 16 h had no significant effect on APP expression, but long-term treatment (6 days) with regular medium change resulted in full rescue of APP gene and protein expression, suggesting an active functional role of ADAM17 in regulation of APP expression (Fig. 3a–c).

### ADAM17 is negatively correlated with APP expression in human brain

**Fig. 4:**
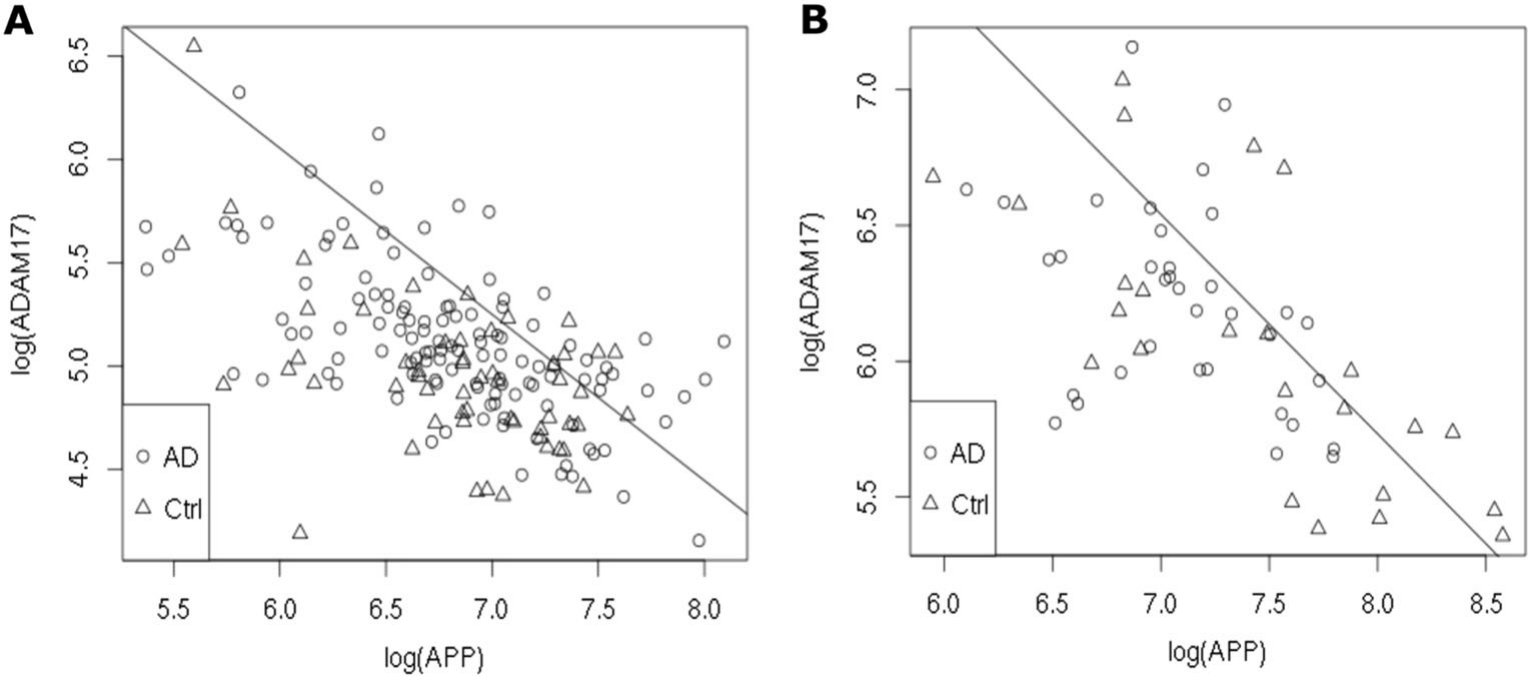
*APP* expression is negatively correlated with *ADAM17* expression in human brain. Average log_2_ of *ADAM17* gene expression against average log_2_ of *APP* gene expression was plotted. **a** NBB sample set; **b** MUC sample set

We next analyzed a possible correlation between *APP* and *ADAM17* gene expression in human brain. Analysis of gene expression data from 284 human prefrontal cortex samples (NBB data: 201 AD patients and 83 controls, Suppl. Table 2) showed a very strong negative correlation (*p* = 2.2×10^−16^, *r*^2^ = 0.55 for the average of *APP* markers ILMN_2404065, ILMN_2404063, and ILMN_1653283 compared to the average of *ADAM17* markers ILMN_1765779 and ILMN_2121068) of *ADAM17* and *APP* mRNA both, in AD and control brains (Fig. 4a). This negative correlation was replicated in an independent sample comprising 57 human temporal cortex samples (MUC data: 33 AD patients and 24 controls, *p* = 5.8×10^−10^, *r*^2^ = 0.44, Suppl. Table 2; Fig. 4b). These results strongly argue for a general functional connection of both genes in human brain which is not restricted to AD patients.

To find out whether *ADAM17* expression might be correlated with expression of its other targets as well, we used the human brain gene expression data set (NBB) to check for possible correlations of *ADAM17* with 71 known ADAM17 targets. Among the analyzed ADAM17 target genes (Supplementary Table 7), *ADAM17* only showed high correlation (defined as Pearson correlation coefficient of ±0.7 to 1) with *PRNP* (−0.77) and *APP* (−0.74). Other AD related targets, such as *TREM2* or *SORL1* only had low to moderate correlation coefficients (Suppl. Table 7).

In addition, we explored possible correlations between *APP* and both other alpha-secretase genes, *ADAM9* and *ADAM10*, using our human brain gene expression data sets. We observed a very weak negative correlation of *ADAM9* with *APP* gene expression (NBB data: *p* = 0.06, *r*^2^ = 0.01; MUC data: *p* = 0.02, *r*^2^ = 0.06; for *ADAM9* marker ILMN_1727524). For *ADAM10*, a weak positive correlation with *APP* expression was observed (NBB data: *p* = 3.3 × 10^−8^, *r*^2^ = 0.09; MUC data: *p* = 2.6 × 10^−4^, *r*^2^ = 0.17; for the average of *ADAM10* markers ILMN_1718946 and ILMN_2148360).

We next evaluated possible correlations between *APP* and *ADAM17* gene expression in brain and non-brain tissues from public databases using the MERAV tool [30]. Again, a significant negative correlation was observed in brain tissues (*r* = −0.27, *p* = 0.00024), whereas in non-brain tissues, no correlation between *ADAM17* and *APP* was present (*r* = 0.0089, *p* = 0.3421).

Together, we found that among known ADAM17 substrates, gene expression of *APP* and *PRNP* showed the highest observed overall correlation with *ADAM17* gene expression in the brain. Both correlations were negative and *APP* gene expression was not significantly correlated with gene expression of *ADAM9* and *ADAM10*.

## Discussion

Using WGS we identified a single rare nonsynonymous variant p.R215I in *ADAM17* shared in all four affected individuals and co-segregating in an extended family with an autosomal-dominant pattern of late-onset AD. We cannot exclude the possibility that other co-segregating variants are involved in AD pathogenesis as well. However, the *ADAM17* variant was the only variant in a so far known AD relevant gene that was also predicted pathogenic by 7 out of 8 prediction programs, conserved by three out of four conservation scores and additionally located within the maximal LOD score region of the pedigree. Subsequent follow-up of this variant in genotyping and whole-exome sequencing data in 5450 additional case/control samples from Germany, the UK and the USA identified five heterozygous variant carriers among AD patients and none within the control subjects. Information derived from genetic population databases revealed an ultra-rare allele frequency among Europeans and absence in non-European populations.

During the functional characterization of the new *ADAM17* variant, we demonstrated failure of ADAM17 maturation causing a loss of alpha-secretase function. Previously, two rare missense mutations of alpha-secretase ADAM10, a close relative of ADAM17, were identified in autosomal-dominant families with AD [8]. Interestingly, as reported here for ADAM17, these ADAM10 mutations were demonstrated to impair pro-domain cleavage as well [31]. Loss of ADAM10 function also resulted in enhanced Aß formation in mice [31].

Focusing on the ADAM17 target APP, we identified a surprising strong negative correlation between *ADAM17* and *APP* gene expression in human brain tissue. Interestingly, a recent report addressing the strongest identified genetic risk factor for sporadic AD so far, Apolipoprotein E (*APOE*), has uncovered a yet unknown differential effect of *APOE*2, *APOE*3, and *APOE*4 variants on APP transcription [32]. These results corroborate a central involvement of APP in AD pathology and shed new light on the role of differential *APP* gene expression in AD pathogenesis.

Using cellular models, we confirmed that over-expression of wild-type ADAM17 caused a reduction of APP gene and protein expression, suggesting a novel mechanism of APP expression regulation by ADAM17. In literature, different pathways are described potentially linking ADAM17 to *APP* transcription. von Rotz et al. demonstrated that AICD regulates transcription of *APP* after being transported to the nucleus; but AICD generated through the alpha-secretase pathway is rather unstable and rapidly degraded [33, 34]. Also, cleavage of signaling receptors such as the p75 neurotrophin receptor or the TGFß-receptor by ADAM17 could potentially influence *APP* transcription [35–38]. However, the exact mechanism has yet to be determined.

In contrast to wild-type ADAM17, over-expression of the R215I version of ADAM17 failed to reduce APP expression and caused elevated Aß levels in vitro. In line with our results, it was previously reported that induction of ADAM17 coincided with downregulation of APP in rat brain and HEK293 cells, and reduced Aß accumulation and AD-like pathology in the Tg2576 mouse model [39–41]. Moreover, decreased ADAM17-mediated alpha-secretase activity was previously shown to promote disease progression in prion disease and AD and to increase accumulation of Aß and pathogenic prions [42]. In cancer cells, APP was demonstrated to regulate *ADAM17* gene expression [43]. Together, these findings suggest that the R215I mutation of *ADAM17* causes loss of alpha-secretase function and consequently elevated Aß production.

Given that over 80 substrates of ADAM17 have been identified so far, the newly identified variant of ADAM17 most probably causes multiple effects that might play a role in AD pathogenesis. For example, ADAM17 has recently been demonstrated to be the main sheddase of TREM2, a transmembrane protein that was identified as AD risk factor by GWAS [2, 44]. However, the exact mechanisms how ADAM17 contributes to AD pathogenesis remain to be analyzed in the future.

Our results corroborate an involvement of the alpha-secretase ADAM17 in AD pathogenesis by identifying a novel rare loss of function variant of this gene. Future large-scale genetic sequencing approaches aiming at the identification and gene-based analyses of *ADAM17* variants are mandatory to fully understand the genetic contribution of ADAM17 to AD.

## Electronic supplementary material

Supplementary Figure 1

Supplementary Figure 2

Supplementary Figure 3

Supplementary Table 1

Supplementary Table 2

Supplementary Table 3

Supplementary Table 4

Supplementary Table 5

Supplementary Table 6

Supplementary Table 7
