# Supplementary material for: A rare loss-of-function variant of ADAM17 is associated with late-onset familial Alzheimer disease
Source: Mol Psychiatry. 2018 Jul 9;25(3):629–39. doi: 10.1038/s41380-018-0091-8 (PMC7042727; doi:10.1038/s41380-018-0091-8)

**Supplementary Figure 1: Shared haplotype blocks.**

Each chromosome in the schematic karyotype is used to represent information abstracted from the shared chromosomal regions between affected individuals. Shared haplotypes blocks were determined using ISCA (Roach et al., 2010). The candidate variant in ADAM17 (depicted in red) lies in a region on chromosome 2 shared by both affected individuals.


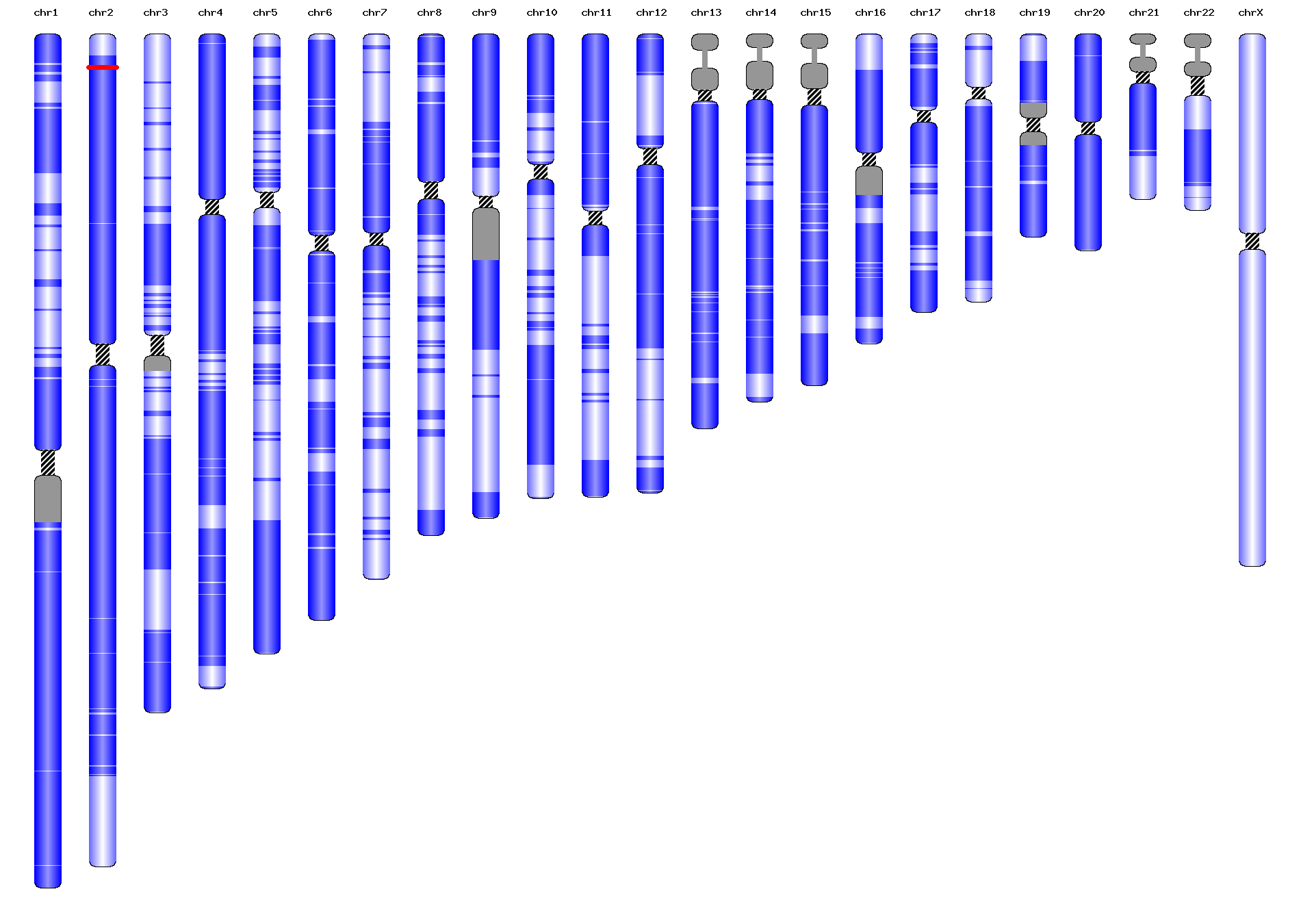

Supplement: Supplementary file 1 — Supplementary Figure 1 [file 41380_2018_91_MOESM1_ESM.docx]
