# Supplementary material for: A rare loss-of-function variant of ADAM17 is associated with late-onset familial Alzheimer disease
Source: Mol Psychiatry. 2018 Jul 9;25(3):629–39. doi: 10.1038/s41380-018-0091-8 (PMC7042727; doi:10.1038/s41380-018-0091-8)
Supplement: Supplementary file 2 — Supplementary Figure 2 [file 41380_2018_91_MOESM2_ESM.docx]

**Supplementary Figure 2: Genetic pedigree analysis.**

**A)** Family genome analysis: schematic filtering strategy. **B)** Pedigree of the family and segregating haplotype blocks. Circles indicate females and squares males. The affected, unaffected and individuals with unknown disease status are filled in black, white and grey, respectively. The ADAM17 variant is marked in red. **C)** Linkage analysis of chromosome 2. The red lines show the upper and lower cut-offs for the LOD score to be considered significant or not being in linkage. The region spanning ADAM17 is marked by dashed lines and connected to **D)**, which shows the region chr2:7341288-11443351. The region in red spans the gene ADAM17, in green the SNPs rs201714573 and rs672354, which are the boundaries of the haplotype block in **B).**
