# Supplementary material for: A rare loss-of-function variant of ADAM17 is associated with late-onset familial Alzheimer disease
Source: Mol Psychiatry. 2018 Jul 9;25(3):629–39. doi: 10.1038/s41380-018-0091-8 (PMC7042727; doi:10.1038/s41380-018-0091-8)
Supplement: Supplementary file 3 — Supplementary Figure 3 [file 41380_2018_91_MOESM3_ESM.docx]

**Supplementary Figure 3:**  **Endogenous APP expression is down-regulated by ADAM17.**

APP gene expression is significantly down-regulated in cells over-expressing ADAM17 wt as compared to control cells (n=3, p(wt)=0.0001). No significant difference of APP expression was observed in cells over-expressing A17 (R215I) as compared to control cells. Asterisks indicate significance. Error bars indicate mean with SD.

**
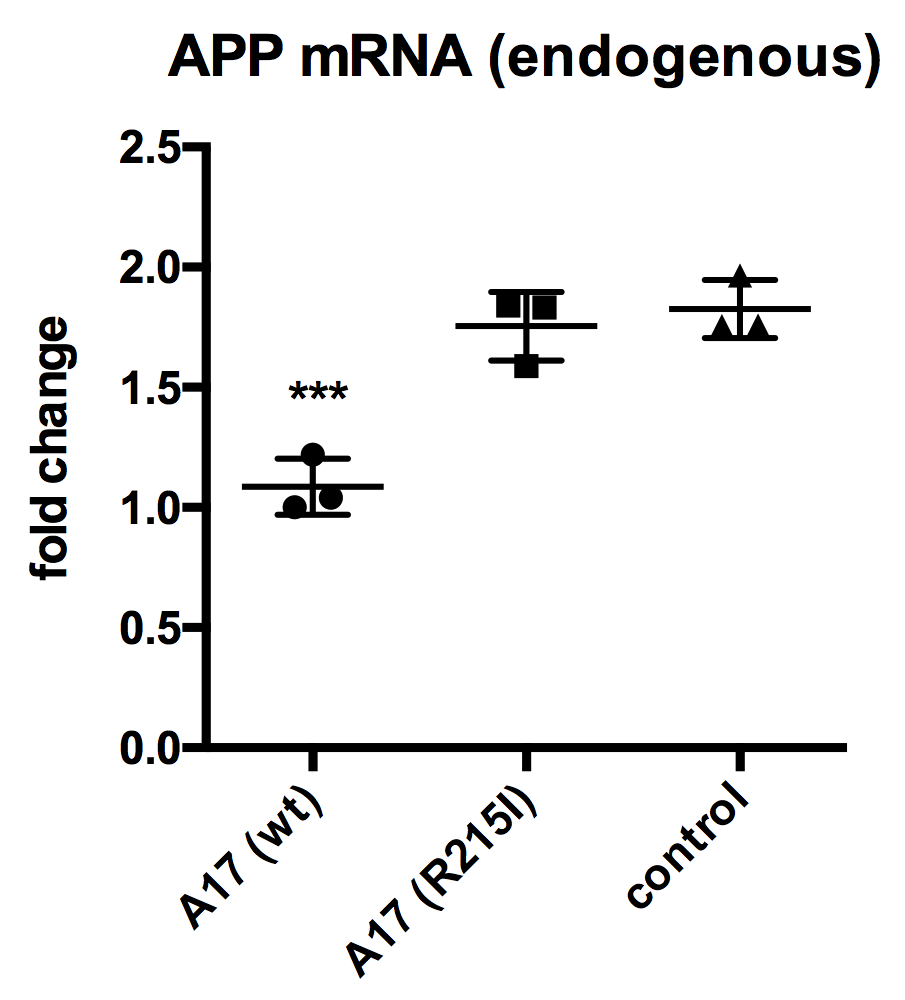
**
