# Supplementary material for: A rare loss-of-function variant of ADAM17 is associated with late-onset familial Alzheimer disease
Source: Mol Psychiatry. 2018 Jul 9;25(3):629–39. doi: 10.1038/s41380-018-0091-8 (PMC7042727; doi:10.1038/s41380-018-0091-8)
Supplement: Supplementary file 4 — Supplementary Table 1 [file 41380_2018_91_MOESM4_ESM.pdf]

Supplementary Table 1: Exonic candidate variants co-segregating with AD in the analyzed family members and likely being pathogenic.

Gene name	chromosome	begin	end	refGene.aachange	ExonicFunc.refGene	SIFT_pred	Polyphen2_HDIV_pred	Polyphen2_HVAR_pred	LRT_pred	MutationTaster_pred	PROVEAN_pred	fathmm-MKL_coding_pred	MetaSVM_pred	DELPREDSUM	DANN_score	CADD_raw	Condel	GERP++_RS	SiPhy_29way_logOdds	DiseaseCausalProb_ExoVarTrainedModel	IsWithinCandidateGene	LODscore	AD-PPI interactions
ADAM17	chr2	9666348	9666349	ADAM17:NM_003183:exon6:c.G644T:p.R215I	nonsynonymous SNV	D	D	P	D	D	D	D	T	7	0.978	4.685	0.506955763834696	3.65	11.203	0.14749289300958188	Y	1,2032	ADAM17<-<br>>MMP14,ADAM17<-<br>>MMP3,ADAM17<-<br>>ACE,ADAM17<-<br>>APP,ADAM17<-<br>>NOTCH1,ADAM17<-<br>>FURIN,ADAM17<-<br>>NOTCH3,ADAM17<-<br>>TIMP2,ADAM17<-<br>>ADAM10,ADAM17<-<br>>NOTCH2,ADAM17<-<br>>SORT1,ADAM17<-<br>>TNFSF11,ADAM17<-<br>>MMP2,ADAM17<-<br>>TIMP1,ADAM17<-<br>>MAPK1,ADAM17<-<br>>NGFR,ADAM17<-<br>>TNFRSF1A,ADAM17<-<br>>CX3CL1,ADAM17<-<br>>BDNF,ADAM17<-<br>>NOTCH4,ADAM17<-<br>>KL,ADAM17<-<br>>TNFRSF1B,ADAM17<-<br>>CXCL12,ADAM17<-<br>>MMP9,ADAM17<-<br>>VEGFA,ADAM17<-<br>>ADAM12,ADAM17<-<br>>ERBB4,ADAM17<-<br>>MMP1,ADAM17<->BACE1
USP34	chr2	61566815	61566816	USP34:NM_014709:exon17:c.G2501T:p.G834V	nonsynonymous SNV	T	D	D	D	D	N	D	T	5	0.996	2.267	0.34250977549096623	5.59	19.589	0.2455167145666326	N	0,0000	
WFS1	chr4	6293694	6293695	WFS1:NM_001145853:exon6:c.G683A:p.R228H,WFS1:NM_006005:exon6:c.G683A:p.R228H	nonsynonymous SNV	D	D	D	D	D	N	D	D	7	1.000	6.846	0.5533741897084593	3.45	12.360	0.13150459923806931	N	0,0000	WFS1<->GSK3A,WFS1<-<br>>FTO,WFS1<-<br>>CDH23,WFS1<-<br>>TCF7L2,WFS1<-<br>>LMNA,WFS1<-<br>>TPP1,WFS1<-<br>>HHEX,WFS1<->ELN
TBC1D14	chr4	6925579	6925580	TBC1D14:NM_001113361:exon2:c.C464T:p.S155F,TBC1D14:NM_020773:exon2:c.C464T:p.S155F	nonsynonymous SNV	D	D	D	D	D	N	D	T	6	0.997	4.829	0.37223323523863044	4.73	16.872	0.21778023875477848	N	0,0000	
GPR125	chr4	22422614	22422615	GPR125:NM_145290:exon12:c.G1703A:p.R568H	nonsynonymous SNV	T	D	D	D	D	N	D	T	5	0.999	6.390	0.48120971734979573	5.1	18.882	0.21275939973079794	N	0.0000	
LIMCH1	chr4	41615578	41615579	LIMCH1:NM_001112719:exon2:c.C121T:p.R41C,LIMCH1:NM_001112720:exon2:c.C121T:p.R41C,LIMCH1:NM_001112717:exon7:c.C583T:p.R195C,LIMCH1:NM_001112718:exon7:c.C583T:p.R195C,LIMCH1:NM_014988:exon7:c.C583T:p.R195C	nonsynonymous SNV	D	D	D	N	D	D	D	T	6	0.999	7.243	0.48866020928314363	4.74	11.427	0.16255421454386143	N	0,0000	
TNIP1	chr5	150416472	1,5E+08	TNIP1:NM_001252386:exon12:c.G1114C:p.E372Q,TNIP1:NM_001258455:exon12:c.G1273C:p.E425Q,TNIP1:NM_001258456:exon12:c.G1273C:p.E425Q,TNIP1:NM_001252385:exon13:c.G1273C:p.E425Q,TNIP1:NM_001252390:exon13:c.G1273C:p.E425Q,TNIP1:NM_001252391:exon13:c.G1273C:p.E425Q,TNIP1:NM_001252392:exon13:c.G1273C:p.E425Q,TNIP1:NM_001252393:exon13:c.G1273C:p.E425Q,TNIP1:NM_001258454:exon13:c.G1273C:p.E425Q,TNIP1:NM_006058:exon13:c.G1273C:p.E425Q	nonsynonymous SNV	T	D	D	D	D	N	D	T	5	0.998	6.136	0.5640015581085525	5.53	19.470	0.19216200000290765	N	0,0000	TNIP1<->TNFAIP3,TNIP1<-<br>>NFKB1
DAAM2	chr6	39869131	39869132	DAAM2:NM_001201427:exon24:c.G2866A:p.E956K,DAAM2:NM_015345:exon24:c.G2863A:p.E955K	nonsynonymous SNV	D	D	P	D	D	D	D	T	7	0.999	5.809	0.49086129330090683	4.63	14.178	0.1829870149983611	N	0,0000	DAAM2<->DVL1,DAAM2<-<br>>CELSR2
DDHD2	chr8	38107282	38107283	DDHD2:NM_001164232:exon11:c.A1306C:p.K436Q,DDHD2:NM_015214:exon11:c.A1306C:p.K436Q	nonsynonymous SNV	D	D	D	D	D	D	D	T	7	0.995	5.304	0.6277892683947199	5.5	15.094	0.306567171610916	N	1,2033	
ADRA2A	chr10	112838908	1,13E+08	ADRA2A:NM_000681:exon1:c.G1155C:p.K385N	nonsynonymous SNV	D	D	D	U	D	D	D	D	7	0.991	4.155	0.6319595917783705	-3.02	11.443	0.15734106894419156	Y	0.0000	[illegible]<br>>SLC6A3,ADRA2A<-<br>>SSTR1,ADRA2A<-<br>>GAL,ADRA2A<-<br>>OPRK1,ADRA2A<-<br>>GALR3,ADRA2A<-<br>>C3,ADRA2A<-<br>>NPY,ADRA2A<-<br>>GHR,ADRA2A<-<br>>DRD4,ADRA2A<-<br>>HEBP1,ADRA2A<-<br>>CXCL1,ADRA2A<-<br>>DRD3,ADRA2A<-<br>>AR,ADRA2A<-<br>>PPYR1,ADRA2A<-<br>>AGTR2,ADRA2A<-<br>>LPAR3,ADRA2A<-<br>>DRD2,ADRA2A<-<br>>PYY,ADRA2A<-<br>>TGFBR2,ADRA2A<-<br>>SSTR2,ADRA2A<-<br>>CCR3,ADRA2A<-<br>>PTGER3,ADRA2A<-<br>>C5AR1,ADRA2A<-<br>>ADRB3,ADRA2A<-<br>>FPR3,ADRA2A<-<br>>FPR2,ADRA2A<-<br>>OPRL1,ADRA2A<-<br>>NPY2R,ADRA2A<-<br>>GALR2,ADRA2A<-<br>>P2RY13,ADRA2A<-<br>>CHRM2,ADRA2A<-<br>>ADRBK1,ADRA2A<-
TSC22D1	chr13	45147304	45147305	TSC22D1:NM_183422:exon1:c.A2906T:p.D969V	nonsynonymous SNV	D	D	D	D	D	D	D	T	7	0.992	4.006	0.4350670955132221	5.16	14.325	0.1777355402267678	N	0.0000	TSC22D1<->TPT1
				evidence for pathogenicity<br>deleterious parameter		D deleteriou	D deleteriou	D deleteriou	D deleteriou	D deleterious	D deleterious	D deleterious	D deleterious	sum of deleteri	DANN>0.9	CADD >! protein disrupting	Condel > 0.5	GERP>3	SiPhy > 10	P>0.3	Yes	LODscore>1	

number of rare (MAF<0.01) exonic and splicing variants segregating with the disease

nonsynonymous	49
synonymous	23
frameshift indel	3
nonframeshift indel	5
splicing	7
