# Supplementary material for: A rare loss-of-function variant of ADAM17 is associated with late-onset familial Alzheimer disease
Source: Mol Psychiatry. 2018 Jul 9;25(3):629–39. doi: 10.1038/s41380-018-0091-8 (PMC7042727; doi:10.1038/s41380-018-0091-8)
Supplement: Supplementary file 5 — Supplementary Table 2 [file 41380_2018_91_MOESM5_ESM.docx]

**Supplementary Table 2: sample characteristics.**

Post-mortem human brain samples from cohorts that have been used in the present study for gene expression correlation analyses.

(a) NBB samples

| **samples** | **number** | **age (mean ± sd) [years]** | **post-mortem delay**  **(mean ± sd) [hours]** | **Braak stage**  **(mean ± sd)** | **APOEε4**  **carrier [%]** |
| --- | --- | --- | --- | --- | --- |
| AD | 200 | 79.29 ± 11.18 | 5.29 ± 1.40 | 5 ± 1 | 66.50 |
| male | 61 | 74.25 ± 11.05 | 5.26 ± 1.30 | 5 ± 1 | 68.85 |
| female | 139 | 81.50 ± 10.53 | 5.30 ± 1.44 | 5 ± 1 | 65.47 |
|  |  |  |  |  |  |
| CTRL | 83 | 81.90 ± 9.35 | 6.74 ± 2.05 | 2 ± 1 | 25.30 |
| male | 35 | 82.23 ± 7.54 | 7.27 ± 2.22 | 2 ± 1 | 22.86 |
| female | 48 | 81.67 ± 10.54 | 6.34 ± 1.83 | 2 ± 1 | 27.08 |

(b) MUC samples

| **samples** | **number** | **age (mean ± sd) [years]** | **post-mortem delay** | **Braak stage** | **APOEε4** |
| --- | --- | --- | --- | --- | --- |
|  |  |  | **(mean ± sd) [hours]** | **(mean ± sd)** | **carrier [%]** |
| AD | 30 | 79.13 ± 8.14 | 29.29 ± 17.43 | 5 ± 1 | 63.33 |
| male | 12 | 77.75 ± 6.94 | 23.80 ± 14.01 | 6 ± 1 | 58.33 |
| female | 18 | 80.06 ± 8.93 | 34.27 ± 19.32 | 5 ± 1 | 66.67 |
|  |  |  |  |  |  |
| CTRL | 22 | 64.68 ± 17.75 | 24.20 ± 10.48 | 1 ± 1 | 13.64 |
| male | 13 | 58.15 ± 17.46 | 23.67 ± 10.14 | 1 ± 1 | 15.38 |
| female | 9 | 74.11 ± 14.15 | 25.00 ± 11.92 | 2 ± 1 | 11.11 |
