# Supplementary material for: A rare loss-of-function variant of ADAM17 is associated with late-onset familial Alzheimer disease
Source: Mol Psychiatry. 2018 Jul 9;25(3):629–39. doi: 10.1038/s41380-018-0091-8 (PMC7042727; doi:10.1038/s41380-018-0091-8)
Supplement: Supplementary file 9 — Supplementary Table 6 [file 41380_2018_91_MOESM9_ESM.docx]

**Supplementary Table 6:** Primer sequences.

| **Primer sequence** | **Description** |
| --- | --- |
| GATCAGGGTACCATGAGGCAGTCTCTCCTATTCCTGACC | KpnI_f |
| GATCAGGGATCCTTAGCACTCTGTTTCTTTGCTGTCAACAC | BamHI_r |
| GTTCTTCATGGGATCTGGGTCAGCTATTCTTTTCACTCGATGAACAAGCTCTTC | 5' ADAM17 p.R215I |
| GAAGAGCTTGTTCATCGAGTGAAAAGAATAGCTGACCCAGATCCCATGAAGAAC | 3' ADAM17 p.R215I |
| TGTTCTGCTCCAAAATTATGTCCCAATGCATGAGTTGTAACCAGGTCAGCTTCCT | 5' ADAM17 p.E406A |
| AGGAAGCTGACCTGGTTACAACTCATGCATTGGGACATAATTTTGGAGCAGAACA | 5' ADAM17 p.E406A |
